# Psychometric Properties of the Positivity Scale among Chinese Adults and Early Adolescents

**DOI:** 10.3389/fpsyg.2018.00197

**Published:** 2018-02-21

**Authors:** Lili Tian, Dandan Zhang, E. Scott Huebner

**Affiliations:** ^1^School of Psychology, Guangdong Key Laboratory of Mental Health and Cognitive Science, Center for Studies of Psychological Application, South China Normal University, Guangzhou, China; ^2^Department of Psychology, University of South Carolina, Columbia, SC, United States

**Keywords:** positive scale (P Scale), factor analyses, reliability, validity, adults, early adolescents

## Abstract

We conducted two studies to explore the psychometric properties of the Positivity Scale (P Scale) among Chinese adults and early adolescents, using a sample of 552 adults (Study 1) and a sample of 888 early adolescents (i.e., middle school students) (Study 2). First, item analyses and factor analyses were conducted to investigate the one-factor structure of the P Scale. Second, internal consistency reliability, test-retest reliability, and external evidences of validity were evaluated to examine its reliability and validity. Last, we used multi-group confirmatory factor analysis to test measurement invariance across gender. The two studies both provided evidence for its reliability and validity among Chinese adults and early adolescents. For the test of measurement invariance across gender, full scalar invariance was established among early adolescents; partial scalar invariance was supported among adults. Taken together, the results provided preliminary support in the Chinese context for the P Scale as a valid measure to assess the general disposition toward viewing life and experiences in a positive manner. The potential applications for future research and professional practice are discussed.

## Introduction

Due to the rise of the positive psychology movement, increasing attention has been paid to positive features of individuals' functioning (Seligman and Csikszentmihalyi, 2000). A large body of research has begun to focus on such positive psychological constructs as self-esteem, optimism, and life satisfaction (Keyes, 2007; Caprara et al., 2010). Although a large number of previous studies has revealed the unique relationships of the three constructs to positive life outcomes, such as health, job success, academic success, and positive interpersonal relationships (Scheier et al., 1994; Baumeister et al., 2003; Lyubomirsky et al., 2005; Nes and Segerstrom, 2006), numerous researchers have been dedicated to the identification of a common latent factor reflecting a general predisposition to view life experiences in a positive manner (Scheier and Carver, 1993; Diener et al., 2000). This basic disposition was initially named “positive thinking” (Caprara and Steca, 2005, 2006), but later named “positive orientation” (Caprara et al., 2009, 2010; Alessandri et al., 2012) or positivity (henceforth labeled POS) (Caprara et al., 2010; Alessandri et al., 2012).

Most notably, research has shown self-esteem, optimism and life satisfaction to all be highly and positively correlated with one another (Diener and Diener, 1995). Caprara and his colleagues (e.g., Caprara and Steca, 2005; Caprara et al., 2009, 2010) focused on what is common to self-esteem, life satisfaction as well as dispositional optimism, and they found a common genetic factor affecting all three constructs, which demonstrated that self-esteem, life satisfaction and optimism were the core features of a trait resembling a basic disposition that was labeled POS.

POS has been corroborated across different cultures (Caprara et al., 2012a). Previous research has suggested that it is quite stable from adolescence through adulthood (Caprara et al., 2012b). Furthermore, previous studies have also shown that POS is a strong predictor of future depression, positive and negative affectivity, happiness, quality of friendships, and health (Caprara et al., 2010; Alessandri et al., 2012; Lauriola and Iani, 2015). Additionally, significant positive associations have been found between POS and other specific positive traits, such as the positive poles of basic personality traits, basic values, self-efficacy beliefs, resilience, trust, pro-sociality, and adjustment in different domains of functioning (Alessandri et al., 2012). Conversely, research has revealed significant negative associations between POS and depression, shyness, hostile rumination, irritability, violence, somatic complaints, and loneliness (Yildiz, 2016). Moreover, POS is an important predictor for many outcomes, for researchers have also shown that there is little variance left to be accounted for by self-esteem, life satisfaction, and optimism once the level of POS is controlled, and when all three constructs are entered simultaneously in one model (Caprara et al., 2010; Alessandri et al., 2012). For instance, recent research found that POS could positively predict future job performance and perceived positive school climate as well (Alessandri et al., 2015; Luengo Kanacri et al., 2017). Finally, researchers have also explored the determinants of POS, with some research demonstrating that extraversion, social support, and a coping style characterized by seeking assistance from others all predict subsequent differences in POS (Lauriola and Iani, 2015; Çevik and Yildiz, 2017).

However, given a lack of suitable measures of POS, latent variable models of POS have been estimated with separately developed measures of self-esteem (Rosenberg, 1965), life satisfaction (Diener et al., 1985) and optimism (Scheier and Carver, 1987) as indicators in past research. In order to assess POS comprehensively with one measure, Caprara and his colleagues developed the *Positivity Scale* (*P Scale*) (Caprara et al., 2012b).

The P Scale was constructed from an initial pool of 36 items. Ultimately, an 8- item scale remained after exploratory and confirmatory factor analyses (CFAs) were interpreted (Caprara et al., 2012b). To date, the *P Scale* has been used in research in America, Canada, Japan, Italy, Germany, Spain, Poland, Serbia, and Brazil. The research has revealed good construct validity, temporal stability, and cross-cultural invariance in Italy, Germany, Canada, and Japan (Caprara et al., 2012a). Also, the findings provide support for the construct validity of the P Scale across Italy, Germany, Spain, Serbia, and Poland (Heikamp et al., 2014). Moreover, research has also demonstrated that the P Scale is a reliable measure with which to evaluate the levels of positivity in Brazil (Borsa et al., 2015). In short, the results of numerous studies have provided evidence of good psychometric properties for the P Scale across multiple countries.

Although the evidence for the psychometric properties of the P Scale has been promising to date, most studies have been done in developed western countries. In order to achieve a more comprehensive understanding of the stability and applicability of the P Scale in different cultural contexts, research on its psychometric properties in other countries and cultures is necessary. As the most populous eastern country, China is one of the largest developing countries in the world, however, research on POS with the P Scale in China remains scarce. One major reason for the limited studies of the POS in China is likely the lack of psychometrically sound measures suitable for Chinese individuals. Considering the urgent need to lay the groundwork for future cross-culture studies and the relevance of evaluating Chinese people's levels of positivity, this study addresses the psychometric properties of the P Scale in the Chinese context.

Due to differences in the cognitive abilities of adults and adolescents (Baltes, 1987), and because the P scale was constructed on the basis of studies of adults, the P Scale may not be as suitable for adolescents as for adults. Therefore, our research was divided into two major studies to examine the generalizability and robustness of the findings for the P Scale for Chinese adults (Study 1) and for early adolescents (Study 2) respectively. In both studies, we explored the factor structure of the P scale first. Subsequently, we examined the internal consistency reliability, test-retest reliability, external evidence of validity, and measurement invariance across gender.

Specifically, we expected a one-factor structure and good internal consistency for the P scale. This expectation was based on previous studies showing that POS can be assessed by estimating the common factor scores of self-esteem, optimism, and life satisfaction (Caprara et al., 2010). External evidence for the concurrent validity of P scale was examined by estimating the associations of the P Scale scores with scores on measures of self-esteem, life satisfaction, optimism, positive and negative affect and loneliness. We also examined the predictive validity of the P scale by estimating the associations of the P scale with scores on measures of depression and subjective well-being 4 weeks later. With respect to self-esteem, life satisfaction, and optimism, we expected they would all be significantly correlated with P scores. Furthermore, consistent with previous research, we expected that P Scale scores would be positively correlated with a measure of positive affect (Caprara et al., 2012b). Additionally, since the P scale is designed to assess individuals' positive orientations toward life experiences whereas negative affect and loneliness represent negative orientations toward life experiences, we expected that the correlation coefficients between the scores on the P scale and measures of negative affect and loneliness would be negative. Regarding predictive validity, we expected POS scores would positively predict later well-being scores and negatively predict later depression scores give that several studies have shown that POS was a significant predictor of measures of positive and negative affectivity (Caprara et al., 2010; Alessandri et al., 2012), Moreover, consistent with findings from other countries, we also expected that the P Scale would show meaningful test-retest reliability and measurement invariance across gender in the Chinese population (Caprara et al., 2012b; Heikamp et al., 2014).

## Study 1

This study addressed the psychometric properties of the Chinese version of the Positivity Scale (P Scale) with Chinese adults.

### Method

#### Participants

A total of 552 adults completed the measures. Participants ranged in age from 18 to 61 years (*M* = 30.25; *SD* = 8.11). A total of 5.4% participants had completed junior high school, 4.6% had completed high school, and 72% had earned a university degree, with 18% completing a master's degree or above. Income ranged from less than 50,000 RMB (10%) to more than 500,000 RMB (3.5%) annually. With regard to marital status, 49.4% of the participants were married; 48.8% were single; 1.2% were divorced and 0.6% were widowed.

The convenience sample was selected from short-term adult education training classes offered at South China Normal University. The study was approved from Human Research Ethics Committee of South China Normal University and the relevant institutional review boards, principals, and training teachers. Participation in the study was voluntary, with written consent required to participate in the study. Six hundred adults consented to participate in the study. Participants were asked to complete the questionnaires on the first and last days of the training, which reflected a 4-week time interval. A series of self-report instruments was administered to the participants by a trained graduate assistant in a regular classroom in the institution. After the participants finished all of the measures, they were debriefed about the purpose of the investigation. Finally, we collected questionnaires from 575 participants and subsequently excluded questionnaires from 23 participants [4.00%; (575–552)/575] because of missing data. Thus, 552 valid respondents were included in the data analyses. Of the 552 participants, 437 participants only completed the first wave of data collection, with 115 participants completing both waves of data collection. Data analyses were performed on the basis of three independent samples (Sample 1, Sample 2, and Sample 3):

**Sample 1:** First, the 437 adults who only participated in the first wave of data collection were randomly split into two halves. The participants in Sample 1 (*N*1 = 219, 64.84% female) included the first half of the adults. Their ages ranged from 19 to 61 (*M* = 32.58, *SD* = 8.99). This sample was used for the descriptive analysis, item analysis, and exploratory factor analysis (EFA).

**Sample 2:** The participants in Sample 2 (*N*2 = 218, 55.96% female) included the other half of the 437 adults, with their ages ranging from 18 to 58 (*M* = 29.33, *SD* = 8.79). This sample was used for the confirmatory factor analysis (CFA), the analyses of internal consistency reliability, relationship with other variables, and measurement invariance.

**Sample 3:** The participants in Sample 3 (*N*3 = 115, 48.70% female) included the adults who participated in both waves of data collection. The age of the participants ranged from 22 to 46 (*M* = 27.57, *SD* = 5.19). This sample was used for the analyses of test-retest reliability and the prediction of depression and well-being from the Positive Scale scores.

#### Measures

##### Positivity scale (P Scale)

The Positivity Scale (P Scale; Caprara et al., 2012b) was designed to directly assess a basic disposition to view life and experiences in a positive manner (POS). In order to obtain the Chinese version, the translation and back translation method was adopted by two different experts who were both proficient in English and Chinese and who did not participate in the study. The method included several steps. First, the scale was translated into Chinese. Second, the Chinese version was back-translated into English by the two independent experts. Following a synthesis of the two versions and minor revisions, the final Chinese version was reviewed by a panel of bilingual psychological experts including the researchers whose first language was English and Chinese. The final Chinese version was compared to the original version to make sure there were no grammatical or semantic issues.

The P Scale consists of eight items. Items 5, 7, and 8 represent an individual's positive view of herself or himself, item 2 represents an individual's satisfaction with life, items 1 and 4 represent an individual's positive attitude about the future, item 3 represents an individual's confidence in other people, and item 6 is a reverse scored item representing an individual's negative view of the future. Participants answered the eight items on a 5-point scale from 1 = *strongly disagree* to 5 = s*trongly agree*. A total score on the P Scale was calculated by summing the item responses to the eight items. Higher scores indicated higher POS.

##### Measures of external evidences of validity

*Self-esteem*. The Rosenberg Self-Esteem Scale (RSES; Rosenberg, 1965; Wang et al., 2010) was used to assess participants' self-esteem, for the Chinese version is used most frequently in current psychological research in China (Tian, 2006). The RSES is a 10-item scale with response options ranging from 1 (strongly disagree) to 4 (strongly agree) (e.g., “I take a positive attitude toward myself”). In the scale, five items are reverse scored item. Scores on the RSES were calculated by summing the item responses to the 10 items, and higher scores indicated higher self-esteem. Previous research has shown that the internal consistency of the scale ranged from 0.72 to 0.89 (Rosenberg, 1965; Corwyn, 2000). In the current study, the Cronbach's alpha coefficient for the RSES was 0.84.

*Life satisfaction*. The Satisfaction with Life Scale (SWLS; Diener et al., 1985) was used to measure participants' global life satisfaction. It is one of the most popular scales in the measurement of life satisfaction (Oishi, 2006). The SWLS is a five-item Likert scale with response options ranging from 1 (*strongly disagree*) to 7 (*strongly agree*) (e.g., “In general, I am satisfied with my life.”). Total scores on the SWLS were calculated by summing the respondents' responses to the five items. Higher scores on the SWLS indicated higher levels of satisfaction. Previous research has revealed that the SWLS demonstrated good reliability and validity with Chinese participants (Xiong and Xu, 2009). In the current study, the Cronbach's alpha coefficient for the SWLS was 0.86.

*Optimism*. The Chinese Revised Life Orientation Test (CLOT-R; Lai et al., 1998) was used to assess optimism. It is an adaptation of the revised LOT (LOT-R; Scheier et al., 1994) for measuring respondents' expectations about the future and a general sense of optimism among Chinese individuals. The CLOT-R involves a 5-point response option scale ranging from 1 (*strongly disagree*) to 5 (*strongly agree*), comprised of six item (e.g., “I am always optimistic about my future”) but three reverse scored items (e.g., “I hardly ever expect things to go my way.”). Total scores on the CLOT-R were calculated by summing the participants' responses to the six items. Higher scores indicated higher levels of optimism. A wide range of studies has examined the utility of the CLOT-R to measure optimism among Chinese persons (Chang et al., 1994; Chang and McBride-Chang, 1996; Lai and Yue, 2000; Li, 2012). In the current study, the Cronbach's alpha coefficient for the total CLOT-R score was 0.63.

*Positive and negative affect*. The Positive and Negative Affect Scale (PANAS; Watson et al., 1988) was used to assess the participants' positive and negative affectivity. The PANAS is comprised of 10 positive emotion terms (e.g., “active”), which reflect positive affectivity (PA), and 10 negative emotion terms (e.g., “afraid”), which reflect negative affectivity (NA). Individuals are asked to report the frequency with which they generally have experienced each emotion, ranging from 1 (*never/almost never*) to 5 (*always/almost always*). Scores for the PA and NA subscales were calculated by summing the particular item response representing for the dimensions of positive and negative affect respectively. Previous research has supported the validity of the PANAS with Chinese individuals (Huang et al., 2003). In the current study, the Cronbach's alpha coefficient was 0.89 for the PA subscale and 0.90 for the NA subscale.

*Subjective well-being*. The Index of Well-being (IWB; Campbell, 1976) was used to assess the degree of subjective well-being of the respondents during the present time period. The measure includes two dimensions of subjective well-being: affect and overall life satisfaction. Eight items tap affect, and one item taps overall life satisfaction. The Response options for life satisfaction ranged from 1 (*extremely dissatisfied*) to 7 (*extremely satisfied*), and affect was rated by pairs of opposite feeling ranging from 1 (i.e., *boring*) to 7 (i.e., *interesting*). Total scores were calculated using the formula: Index = 1.1 × (overall life satisfaction item score) + 1.0 × (mean of eight general affect scores). Total scores ranged from 2.10 to 14.70, and higher scores indicated higher levels of subjective well-being. Previous research has shown that the IWB is psychometrically sound for use with Chinese adults (Jing, 2000). In this study, the Cronbach's alpha coefficient was 0.89.

*Depression*. The Center for Epidemiologic Studies Depression Scale (CES-D; Fava, 1983) was used to measure depressive symptoms. The CES-D involves a 4-point Likert scale rated from 1 [*rarely or none of the time* (less than 1 day)] to 4 [*most or all of the time* (5–7 days)] according to the frequency of occurrence on the 20 specific symptoms (e.g., “I was bothered by things that usually don't bother me”). Scores on the CES-D were calculated by summing all the responses to the items, and higher scores indicated higher levels of depression. Evidence has been provided to support the validity of CES-D for use with Chinese adults (Zhang et al., 2010). In this study, the Cronbach's alpha coefficient was 0.92.

#### Data analysis

Descriptive statistics were calculated for the eight items of the P Scale as well as the mean score of the P Scale. Corrected item-total correlations for the eight items of the P Scale were used for the item analysis. The factor structure examination of the P Scale was conducted by EFAs and CFAs. We chose Chi square (χ^2^), the comparative fit index (CFI), the Tucker-Lewis index (TLI), the standardized root mean square residual (SRMR) and the root mean square error of approximation (RMSEA) to assess model fit. The criteria employed for an acceptable model fit were the following: CFI > 0.90, TLI > 0.90, SRMR ≤ 0.08, RMSEA < 0.10. The criteria for a good model fit were the following: CFI > 0.95, TLI > 0.95, SRMR ≤ 0.04, RMSEA < 0.06 (Bentler, 1990; Browne and Cudek, 1993; Schreiber et al., 2006). Cronbach's alpha coefficient was used to measure the internal consistency for the eight items of the P Scale. Aiming to measure the concurrent relationships between POS and other variables, Pearson correlations were calculated between the mean score of the P Scale and scores on the RSES, SLWS, PA, NA, and CLOT-R. In regard to the prediction of subjective wellbeing and depression from POS, hierarchical multiple regression analyses were conducted using the P Scale scores at Time 1 as the predictor variable and the scores on the IWB and CES-D at Time 2 as the criterion variables. Also, the Pearson correlation between the scores of the P Scale at Time 1 and Time 2 was calculated to examine the test-retest reliability over a 4-week period. Finally, we used multi-group confirmatory factor analysis to assess the measurement invariance of the P Scale across gender. CFAs, EFAs and measurement invariance testing were computed using Mplus 6.0 (Muthen and Muthen, 2011), and the other data analysis procedures were conducted using SPSS 16.0 (SPSS Inc., Chicago, IL, USA).

### Results

#### Descriptive analysis and item analysis

The means, standard deviations, skewness, kurtosis, corrected item-total correlations, and the correlations of the eight items of the P scale for Sample 1 are shown in Table 1. The mean score for the eight items ranged from 2.64 to 3.87, the skewness of all the items ranged from −1.15 (Item 5) to 0.52 (Item 6) and the kurtosis ranged from −0.56 (Item 6) to 1.74 (Item 5), revealing that the scores on variables reflected a reasonably normal distribution. After reverse scoring (Item 6), all the eight items significantly positive correlated with each other, and the corrected item-total correlations for the eight items of the P Scale ranged from 0.47 (Item 6) to 0.72 (Item 4). Together, these results provided support to further explore the factor structure of the P scale with Sample 1.

**Table 1:** Means, standard deviations, skewness, kurtosis, the corrected item-total correlations, the correlations of the eight items of the P scale, and factor loading for Chinese adults (Sample 1, *N* = 219; Sample 2, *N* = 218).

**Variable**	**1**	**2**	**3**	**4**	**5**	**6**	**7**	**8**	**M**	**SD**	**Skew**	**Kurt**	**Factor loading**	**CITC**
**SAMPLE 1 (*N* = 219)**														
1.Item1	–								3.87	0.76	−0.46	0.43	0.77	0.68
2.Item2	0.59^**^	–							3.60	0.85	−0.56	−0.17	0.79	0.70
3.Item3	0.39^**^	0.47^**^	–						3.44	0.81	−0.45	0.19	0.65	0.53
4.Item4	0.71^**^	0.61^**^	0.47^**^	–					3.83	0.73	−0.75	1.65	0.82	0.72
5.Item5	0.52^**^	0.59^**^	0.44^**^	0.63^**^	–				3.78	0.73	−1.15	1.74	0.78	0.68
6.Item6	0.45^**^	0.43^**^	0.28^**^	0.38^**^	0.34^**^	–			2.64	1.11	0.52	−0.56	0.58	0.47
7.Item7	0.40^**^	0.48^**^	0.37^**^	0.46^**^	0.51^**^	0.28^**^	–		3.61	0.79	−0.56	0.47	0.70	0.59
8.Item8	0.41^**^	0.40^**^	0.39^**^	0.43^**^	0.47^**^	0.34^**^	0.59^**^	–	3.68	0.75	−0.69	0.66	0.68	0.58
POS	0.76^**^	0.79^**^	0.65^**^	0.79^**^	0.76^**^	0.65^**^	0.69^**^	0.68^**^	3.56	0.59	−0.31	3.56		
**SAMPLE 2 (*N* = 218)**														
1.Item1	1								3.89	0.70	−0.30	0.20	0.61	
2.Item2	0.41^**^	1							3.45	0.86	−0.59	−0.37	0.72	
3.Item3	0.35^**^	0.44^**^	1						3.34	0.87	−0.26	−0.33	0.57	
4.Item4	0.66^**^	0.47^**^	0.41^**^	1					3.81	0.70	−0.92	1.69	0.70	
5.Item5	0.49^**^	0.63^**^	0.45^**^	0.58^**^	1				3.73	0.80	−0.90	0.73	0.80	
6.Item6	0.26^**^	0.27^**^	0.14^*^	0.22^**^	0.22^**^	1			2.63	0.97	0.53	−0.26	0.33	
7.Item7	0.38^**^	0.49^**^	0.35^**^	0.43^**^	0.52^**^	0.27^**^	1		3.57	0.79	−0.44	0.03	0.68	
8.Item8	0.47^**^	0.39^**^	0.37^**^	0.50^**^	0.47^**^	0.25^**^	0.56^**^	1	3.61	0.80	−0.56	0.08	0.65	
POS	0.70^**^	0.74^**^	0.64^**^	0.75^**^	0.78^**^	0.50^**^	0.72^**^	0.71^**^	3.50	0.56	−0.17	0.26		

*M, Mean; SD, Standard deviation; Skew, Skewness; Kurt, Kurtosis; POS, Mean score of positivity; CITC, Corrected item-total correlations*.

* *p < 0.05*,

** *p < 0.01*.

#### Factor analysis

##### Exploratory factor analysis

The results of the Bartlett's test (χ^2^ = 731.98, *df* = 28, *p* < 0.001) and the Kaiser-Meyer-Olkin statistic (KMO = 0.88 > 0.5) both showed that the data were suitable for EFA. Principal component analysis was adopted to perform EFAs. The first five eigenvalues were 4.24, 0.87, 0.73, 0.64 and 0.48, which supported the one-factor structure, for the ratio of the first to the second eigenvalues was higher than 2 (i.e., 4.87; Hattie, 1985). In addition, we adopted Mplus to conduct EFA as well, choosing the standardized root-mean-square residual (SRMR) as the index of goodness of fit. Hu and Bentler (1998) suggested that values higher than 0.08 indicated poorer fit to the empirical data, whereas values lower than 0.05 suggested an excellent fit. The SRMR of the one factor model was 0.046, fitting the data well. The factor accounted for 52.95% of the variance. Factor loadings of the model ranged from 0.58 to 0.82 (see Table 1).

##### Confirmatory factor analysis

Because different factorial structures may emerge for different samples (Kline, 2005), we conducted CFA procedures with Sample 2 by adopting the maximum likelihood estimation to examine the replicability of the resulting factor structure from the EFA with Sample 1. As shown in Table 1, the skewness and kurtosis of all the variables were well below the most commonly used critical values for univariate normality (±3 and ±8; Kline, 1998), revealing that the distribution of all the items was fairly normal. The results of the CFA supported the one-factor model, revealing an acceptable fit: $\chi_{\left(20,\;\text{N}=218\right)}^{2}$ = 67.25, *p* < 0.001; CFI = 0.923; TLI = 0.892; RMSEA = 0.104, 90% CI [0.077, 0.132]; SRMR = 0.044. However, the modification indexes analysis showed that the correlation of one error item pair (items 1 and 4) would significantly decrease the chi square value, and consequently increase the RMSEA. Considering that the content of item 1 (i.e., “I have great faith in the future.”), and item 4 (i.e., “I look forward to the future with hope and enthusiasm.”) both concerned positive attitudes toward the future, we allowed the residuals of the item pairs to be correlated. The revised model yielded excellent fit indices: $\chi_{\left(19,\;\text{N}=218\right)}^{2}$ = 37.09, *p* < 0.01; CFI = 0.971; TLI = 0.957; SRMR = 0.035; RMSEA = 0.066, 90% CI [0.033, 0.098]. Factor loadings for the model ranged from 0.33 (Item 6) to 0.80 (Item 5) (*M* = 0.63, *SE* = 0.05).

#### Internal consistency and test-retest reliability

The Cronbach's alpha coefficients (sample 1: α = 0.86; sample 2: 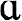 = 0.84; sample 3: 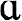 = 0.84) for the three samples and the 4-week test-retest reliability coefficient (*r* = 0.76) for Sample 3 together suggested good internal consistency reliability and temporal stability for the P Scale for Chinese adults.

#### External evidences of validity

##### Relationships with other variables

Table 2 showed that the P Scale correlated positively with the RSES (*r* = 0.70, *p* < 0.01), SWLS (*r* = 0.62, *p* < 0.01), CLOT-R (*r* = 0.47, *p* < 0.01), PA (*r* = 0.49, *p* < 0.01) and correlated negatively with NA (*r* = −0.38, *p* < 0.01). As expected, the P Scale correlated positively with RSES, SWLS, CLOT-R and the scale measuring positive affectivity whereas it correlated negatively with the scale measuring negative affectivity. Together, the results provided support for external evidence of validity of the P Scale among Chinese adults.

**Table 2:** Pearson correlations among the P scale, RSES, SWLS, C-LOT, PA, and NA for Chinese adults *(N* = 218).

**Constructs**	**P Scale**	**RSES**	**SWLS**	**C-LOT**	**PA**	**NA**
Positivity Scale (P Scale)						
Self-esteem (RSES)	0.70^**^					
Life satisfaction (SWLS)	0.62^**^	0.46^**^				
Optimism (C-LOT)	0.47^**^	0.47^**^	0.24^**^			
Positive affect (PA)	0.49^**^	0.47^**^	0.30^**^	0.26^**^		
Negative affect (NA)	−0.38^**^	−0.47^**^	−0.29^**^	−0.34^**^	−0.03	

** *p < 0.01*.

##### Prediction of well-being and depression

Hierarchical multiple regression analyses were applied to estimate the predictive validity of the P scale by assessing the relations between the scores on the P Scale at Time 1 and the scores on the IWB and CES-D at Time 2. Before conducting the regression analyses, we evaluated the multicollinearity of variables via the variance inflation factor (VIF), which is a measure of the degree of multicollinearity in a set of multiple regression variables; values >10 indicate collinearity (Marquardt, 1970). In the present study, issues with multicollinearity did not appear to be germane given that the VIF was well below the criterion level (VIF = 1.04). Thus, it was considered appropriate to perform the subsequent hierarchical multiple regression analyses.

In order to investigate the contribution of the P Scale to the prediction of subsequent subjective well-being and depression scores, the scores on the P Scale at Time 1 were employed as the predictor variable, and the scores on the IWB and CES-D at Time 2 served as the criterion variables. First, correlated demographic variables (including gender and age) were entered as the initial block of control variables, and then, scores on the P Scale were entered as the second block of predictor variables. The categorical variable (Gender) was dummy coded as 0 = males, 1 = females before being entered in the regression analysis. As shown in Table 3, the overall model accounted for 27.3% and 31.2% of the variance in subjective well-being and depression respectively, and the scores of the P scale at Time 1 made a significant, positive contribution to subjective well-being (β = 0.47, *p* < 0.001, Δ*R*^2^ = 0.208), and a negative contributions to depression (β = −0.53, *p* < 0.001, Δ*R*^2^ = 0.275) at Time 2. The results thus showed that POS scores were a significant predictor of subjective well-being and depression 4 weeks later among Chinese adults, after controlling for the influence of gender and age.

**Table 3:** Hierarchical multiple regression of subjective wellbeing and depression at time 2 on the total score of the P scale at Time 1 (Sample 3, *N* = 115).

	***B***	**SE**	**β**	***R*^2^**	**Δ*R*^2^**	**Δ*F***	***df***
**SUBJECTIVE WELL BEING**							
Step 1				0.065	0.065	3.90^*^	(2, 112)
Intercept	8.99	1.26					
Gender^a^	1.24	0.44	0.26^**^				
Age	0.02	0.04	0.04				
Step 2				0.273	0.208	31.76^***^	(1, 111)
Intercept	3.56	1.47					
Gender^a^	1.03	0.39	0.21				
Age	−0.02	0.04	−0.04				
T1 P scale	0.23	0.04	0.47^***^				
**DEPRESSION**							
Step 1				0.037	0.037	2.13	(2, 112)
Intercept	19.32	4.62					
Gender^a^	−3.18	1.62	−0.19				
Age	−0.16	0.16	−0.10				
Step 2				0.312	0.275	44.35^***^	(1, 111)
Intercept	41.90	5.19					
Gender^a^	−2.32	1.39	−0.14				
Age	0.01	0.14	0.004				
T1 P scale	−0.97	0.15	−0.53^***^				

*P Scale, Positivity scale; T1 P Scale, the total score of the P Scale at Time 1*.

a 0, Males; 1, Females;

* *p < 0.05*,

** *p < 0.01*,

*** *p < 0.001*.

##### Measure invariance of the P Scale

Because the result of the maximum likelihood estimator (MLR) can be influenced by the sample size, we conducted a series of stepwise analyses by adopting the robust MLR for the assessment of measurement invariance to determine the extent to which P Scale items reflect comparable meanings across gender. This kind of estimator will correct and adjust the statistics and standard error to improve the accuracy of the result. Correspondently, it will yield the scaled chi-square test statistic (S-Bχ^2^) as the model indicator (Finney and Distefano, 2013). Given that the residuals of item 1 and item 4 correlated with each other in the previous analysis, we allowed the residuals of items 1 and 4 to correlate in the assessment of measurement invariance as well. Three levels of measurement invariance analyses were tested in the following order: Configural invariance, metric invariance, and scalar invariance. Configural invariance examines the equivalence of the factor structure across groups; metric invariance examines the equivalence of the factor loadings across groups (Vandenberg and Lance, 2000), and scalar invariance analysis examines the equivalence of the factor loadings and item intercepts across groups (Rensvold and Cheung, 2001).

To test for invariance in the P Scale, baseline CFAs were conducted first for each group (men and women) separately. For men, the indices were S-B$\chi_{\left(19,\;\text{N}=96\right)}^{2}$ = 29.21, *p* > 0.01; CFI = 0.950; TLI = 0.926; RMSEA = 0.075, 90% CI [0.000, 0.126]; SRMR = 0.055, and for women, the indices were S-B$\chi_{\left(19,\;\text{N}=122\right)}^{2}$ = 31.04, *p* > 0.01; CFI = 0.958; TLI = 0.939; RMSEA = 0.072, 90% CI [0.016, 0.116]; SRMR = 0.044. In both samples, the one-factor model resulted in an adequate fit. Next, two-group CFAs were conducted to test for the comparability of the P Scale between men and women. The CFI difference test (ΔCFI) between the configural invariance model and the metric invariance model was 0.002, which was within the cutoff score of ΔCFI ≤ 0.01 (Rensvold and Cheung, 2001). The Satorra-Bentler different test (ΔS-Bχ^2^; Satorra and Bentler, 1994) was ΔS-B$\chi_{\left(7\right)}^{2}$ = 4.54, *p* > 0.01. Thus, metric invariance across gender was supported. Comparing the metric invariance model and the scalar invariance model, the indices were ΔCFI = −0.063; ΔS-B$\chi_{\left(7\right)}^{2}$ = 34.53, *p* > 0.01, which demonstrated that full scalar invariance was not supported. We then tested for partial scalar invariance using a backward method by removing the constraints that contributed more chi-square values to the model until the partial scalar invariance model was acceptable. Finally, a model for partial scalar invariance(ΔCFI = −0.002; ΔS-B$\chi_{\left(4\right)}^{2}$ = 4.86, *p* > 0.01) across the two gender groups was retained after relaxing the constraints on the intercepts of three items (i.e., “Item 2, Item 3, Item 5”). The fit of all the models is shown in Table 4.

**Table 4:** Analysis of measurement invariance across gender in adults (Sample 2, *N* = 218).

**Model**	**S-Bχ^2^**	***df***	**CFI**	**TLI**	**RMSEA**	**SRMR**		**ΔS-Bχ^2^(Δ*df*)**	**Δ CFI**
Man	29.21	19	0.950	0.926	0.075	0.055			
Women	31.04	19	0.958	0.939	0.072	0.044			
M1:Configural invariance	60.56	38	0.955	0.934	0.074	0.049			
M2:Metric invariance	66.78	45	0.957	0.946	0.067	0.066	M1:M2	4.54(7)	0.002
M3:Scalar invariance	104.11	52	0.894	0.885	0.112	0.112	M2:M3	34.53(7)	−0.063
M4:Partial scalar invariance	71.83	49	0.955	0.948	0.065	0.068	M4:M2	4.86(4)	−0.002

*ΔS-Bχ^2^, the Satorra-Bentler scaled (mean-adjusted) Chi-square difference between nested models; Δdf, Difference in degrees of freedom between nested models; ΔCFI, Comparative fit index differences between nested models*.

## Study 2

This study aimed to test whether the P Scale also showed good psychometric properties in Chinese early adolescents. The findings of Study 1, which provided support for the psychometric properties of the P Scale with Chinese adults, offered a strong foundation to further examine whether the P Scale is suitable to evaluate the positivity disposition of Chinese early adolescents as well. Therefore, in this study, we investigated the psychometric properties of the P Scale with Chinese middle school students.

### Method

#### Participants

The participants were 888 middle school students (440 girls and 448 boys) ranging in age from 11 to 15 years (*M* = 12.97, *SD* = 0.67). We drew the convenience sample from public schools located in a northern China province. According to the information given by the local education authorities, these schools were typical, coeducational schools, they were all reasonably representative of schools in that province. All of the schools were comparable in terms of the students' academic performance, school size, average class size, and teachers' teaching ability. A total of 69.1% of the participants' fathers earned at least a high school diploma, and 93.8% had a steady career. A total of 67.0% of the participants' mothers earned at least a high school diploma, and 81.4% having a steady career.

The procedures for this investigation were the same as those for the adult participants in Study 1. All students participated voluntarily in the two waves of data collection. Written parent consent and student assent were both required to participate in this study. Although 924 students consented to participate in the study, we collected 902 students' questionnaires. Students [1.5%; (902–888)/902] whose questionnaires had any missing values were excluded from the study. The final sample included 888 valid respondents, in which 694 students completed only the first one wave of data collection, and 194 students completed both waves of data collection (across 6 months). In the current study, data analyses were conducted based on three independent middle school student samples (Sample 1, Sample 2, and Sample 3).

**Sample 1**: Initially, all of the middle school students participating in the first wave of data collection were randomly split in two halves. Sample 1 (*N*1 = 350, 52.00% girls) included the first half of the total middle school student sample, with ages ranging from 10 to 15 (*M* = 12.96, *SD* = 0.69). The sample was used for item analysis and EFA.

**Sample 2**: Sample 2 (*N2* = 344, 50.58% girls) included the other half of the students participating in the first wave of data collection, with ages ranging from 10 to 15 (*M* = 12.98, *SD* = 0.67). This sample was used for the CFA, analyses of internal consistency reliability, relationship with other variables, and measurement invariance testing.

**Sample 3**: This sample (*N3* = 194, 43.30% girls) included the middle school students who participated during both Wave 1 and Wave 2 (6 months later), with ages ranging from 11 to 15 (*M* = 12.97, *SD* = 0.63). This sample was used for the analyses of test-retest reliability and the prediction of depression and well-being from P scale scores.

#### Measures of external evidences of validity

##### Self-esteem

The Rosenberg Self-Esteem Scale (RSES; Rosenberg, 1965) was used to investigate the middle school students' self-esteem. Previous research has shown good psychometric properties of RSES when used with Chinese middle school students (Dang et al., 2016). In the present study, the Cronbach's alpha coefficient of the RSES was 0.81.

##### Life satisfaction

The Brief Multidimensional Students' Life Satisfaction Scale (BMSLSS; Seligson et al., 2003) was adopted to assess the life satisfaction of Chinese middle school students. The Chinese version of the BMSLSS consists of six items, with five items used to measure children and adolescents' satisfaction with family, friends, schools, self and living environment. (e.g., “I would describe my satisfaction with my family as….). One additional item was used to measure global or overall life satisfaction. The BMSLSS employs a seven-point response option scale with options ranging from 1 (*terrible*) to 7 (*delighted*). Scores were calculated by summing the item responses to the five items and dividing by five. Higher scores indicated higher general life satisfaction. Evidence has suggested good psychometric properties when it has been used with Chinese adolescents (Kwan, 2007; Ye et al., 2013). In the present study, its Cronbach's alpha coefficient was 0.77.

##### Loneliness

The University of California at Los Angeles Loneliness Scale (UCLA; Russell et al., 1978) was adopted to assess the loneliness of Chinese middle school students. The scale was used for evaluating the loneliness caused by the gap between desire for social interaction and the actual level of social interaction. The scale consists of 20 items with response options ranging from 1 (never) to 4 (always) (e.g., “Do you often feel you are lacking partners? friends?”). There were nine reverse scored items in the scale. Total scores on the UCLA were calculated by summing the respondents' responses to the 20 items. Higher scores indicated higher levels of loneliness. Previous research has revealed that the UCLA demonstrated good reliability and validity with Chinese adolescents (Jiang et al., 2017; Sun et al., 2017). In the current study, the Cronbach's alpha coefficient for the UCLA was 0.90.

##### Subjective well-being in school

Brief Adolescents' Subjective Well-Being in School Scale (BASWBSS; Tian et al., 2015) was used to measure the middle school students' subjective well-being in school. The BASWBSS is an 8-item self-report scale including two subscales: School Satisfaction (SS) and Affect in School (AS). The SS subscale consists of six items (e.g., “The teachers' instructional methods and quality are good.”). Items were rated on a 6-point scale, with response options ranging from 1 (*strongly disagree*) to 6 (*strongly agree*). The SS subscale score was calculated through averaging the responses to the six items. The AS subscale consists of two items, which respectively assesses the frequency of positive affect (PA) and negative affect (NA) in school. The AS subscale score was calculated through subtracting the NA from the PA score. Finally, a total BASWBSS score was calculated by summing the SS and AS subscale scores. Previous research has provided empirical support for the SWB in school model and the BASWBSS in Chinese adolescents (Tian et al., 2015). In the present study, the Cronbach's alpha coefficient for the SS subscale was 0.79.

##### Depression

The Depression Self-Rating Scale for Children (DSRSC; Linyan et al., 2003) was used to assess depression. The scale was first compiled by Birleson (1981) to diagnose depressive disorders in children of ages 6 to 18. The DSRSC employs a 3-point response option scale, with options ranging from 0 (*never*) to 2 (*often*). The DSRSC is comprised of 18 items (e.g., “I wanted to run away from home.”), including 10 reverse scored items (e.g., “I have confidence in myself.”). Total scores were calculated by summing across all the item scores, with higher scores reflecting higher levels of depression. Previous work has supported the validity of the DSRSC with Chinese school students (Linyan et al., 2003). In the present study, its Cronbach's alpha coefficient was 0.83.

#### Data analysis

The data analysis procedures were identical to those of Study 1, including descriptive analyses, analyses of factor structure, reliability and validity, and measurement invariance across gender.

### Results

#### Descriptive analyses and item analyses

The descriptive analyses for Sample 1 are shown in Table 5. The mean score for the items ranged from 2.70 (Item 2) to 4.28 (Item 6). After reverse scoring (Item 6), all eight items significantly positive correlated with each other. The indices of skewness and kurtosis showed that all the items were reasonably normally distributed, with the skewness values ranging from−1.03 (Item 4) to 0.23 (Item 6), and the kurtosis values ranging from −0.95 (Item 6) to 1.24 (Item 2) respectively. The corrected item-total correlations for the eight items of the P Scale ranged from 0.33 (Item 6) to 0.64 (Item 4). Similar to the results of Study 1, the cumulative findings support the subsequent exploration of the factor structure of the P scale among middle school students.

**Table 5:** Means, standard deviations, Skewness, Kurtosis, the corrected item-total correlations, the correlations of the eight items of the P scale, and factor loading for Chinese early adolescents (Sample 1, *N* = 350; Sample 2, *N* = 344).

**Variable**	**1**	**2**	**3**	**4**	**5**	**6**	**7**	**8**	***M***	***SD***	**Skew**	**Kurt**	**Factor loading**	**CITC**
**SAMPLE 1 (*N* = 350)**														
1.Item1	–				-				4.08	0.85	−0.83	0.75	0.75	0.62
2.Item2	0.53^**^	–							4.28	0.77	−1.03	1.24	0.72	0.59
3.Item3	0.37^**^	0.42^**^	–						3.64	0.99	−0.53	0.12	0.64	0.51
4.Item4	0.71^**^	0.52^**^	0.45^**^	–					4.10	0.90	−0.98	0.98	0.77	0.64
5.Item5	0.40^**^	0.42^**^	0.37^**^	0.52^**^	–				3.84	0.91	−0.70	0.44	0.76	0.63
6.Item6	0.31^**^	0.18^**^	0.18^**^	0.26^**^	0.24^**^	–			2.70	1.27	0.23	−0.95	0.44	0.33
7.Item7	0.37^**^	0.33^**^	0.33^**^	0.40^**^	0.45^**^	0.13^**^	–		3.62	0.98	−0.47	−0.01	0.59	0.46
8.Item8	0.54^**^	0.42^**^	0.34^**^	0.51^**^	0.49^**^	0.24^**^	0.51^**^	–	3.75	0.97	−0.61	0.13	0.71	0.60
POS	0.77^**^	0.67^**^	0.63^**^	0.78^**^	0.72^**^	0.52^**^	0.64^**^	0.74^**^	3.75	0.64	−0.47	0.29		
**SAMPLE 2 (*N* = 344)**														
1.Item1	–								4.08	0.91	−0.99	1.11	0.76	
2.Item2	0.56^**^	–							4.29	0.73	−0.83	0.66	0.62	
3.Item3	0.44^**^	0.36^**^	–						3.66	0.97	−0.56	0.26	0.54	
4.Item4	0.77^**^	0.54^**^	0.46^**^	–					4.13	0.89	−1.08	1.40	0.79	
5.Item5	0.45^**^	0.38^**^	0.35^**^	0.54^**^	–				3.85	0.91	−0.69	0.46	0.67	
6.Item6	0.35^**^	0.21^**^	0.18^**^	0.29^**^	0.25^**^	–			2.66	1.23	0.22	−0.89	0.36	
7.Item7	0.42^**^	0.35^**^	0.35^**^	0.46^**^	0.48^**^	0.18^**^	–		3.66	0.99	−0.54	0.03	0.64	
8.Item8	0.58^**^	0.41^**^	0.36^**^	0.57^**^	0.54^**^	0.26^**^	0.57^**^	–	3.78	0.95	−0.64	0.23	0.76	
POS	0.81^**^	0.65^**^	0.62^**^	0.82^**^	0.71^**^	0.53^**^	0.68^**^	0.77^**^	3.77	0.66	−0.65	0.88		

*M, Mean; SD, Standard deviation; Skew, Skewness; Kurt, Kurtosis; POS, Mean score of positivity; CITC, Corrected item-total correlations*.

** *p < 0.01*.

#### Factor analyses

##### Exploratory factor analyses

Consistent with the results of Study 1 with adults, the results of the Bartlett's test and the Kaiser-Meyer-Olkin statistic(χ^2^ = 872.1, *df* = 28, *p* < 0.001; KMO = 0.86 > 0.5) both met the criterion for conducting an EFA with the early adolescent students. Thus, a principal component analysis was adopted to perform EFAs. For these middle school students (Sample 1), the first five eigenvalues were 3.72, 0.92, 0.82, 0.74, and 0.52, which supported the one-factor structure, for the ratio of the first to the second eigenvalues was higher than 2 (i.e., 4.04; Hattie, 1985). In addition, the result of the index of goodness of fit (SRMR = 0.044) also support the one factor model. The first factor accounted for 46.44% of the variance, with the factor loadings ranging from 0.44 to 0.77 (see Table 5).

##### Confirmatory factor analysis

According to the values for skewness and kurtosis as shown in Table 5, all the variables reflected univariate normal distributions, with all the values below the critical values (Kline, 1998). Similar to the findings of Study 1, the results of the CFA for Sample 2 also supported the one-factor model suggested by the results of the EFA with Sample 1. The model fit was acceptable as well: $\chi_{\left(20,\;\text{N}=344\right)}^{2}$ = 88.92, *p* < 0.001; CFI = 0.935; TLI = 0.910; SRMR = 0.042; RMSEA = 0.100, 90% CI [0.079, 0.122]. Modification indices indicated that the ill fit of the model was mainly caused by the error covariance of items 1 and 4. After revisions, the model suggested a better fit to the data: $\chi_{\left(19,\;\text{N}=344\right)}^{2}$ = 60.16, *p* < 0.001; CFI = 0.961; TLI = 0.943; SRMR = 0.035; RMSEA = 0.079, 90% CI [0.057, 0.102]. Factor loadings for the model were high, ranging from 0.36 to 0.79 (*M* = 0.64, *SD* = 0.04).

#### Internal consistency and test-retest reliability

The Cronbach's alpha (sample 1: 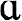 = 0.81; sample 2: 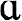 = 0.86; sample 3: 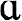 = 0.82) for the three samples and the 6-months test-retest reliability coefficient (*p* = 0.55) for Sample 3 together revealed that the P Scale showed good internal consistency and test-retest reliability for middle school students.

#### External evidence of validity

##### Relationships with other variables

For middle school students, scores on the P Scale correlated positively with the scores on the RSES (*r* = 0.60, *p* < 0.01), the one item global life satisfaction measure (BMSLSS-Global) (r = 0.42, *p* < 0.01) and the five item general life satisfaction(BMSLSS-General) (*r* = 0.44, *p* < 0.01). In addition, the P Scale scores negatively correlated with the UCLA scores (*r* = −0.60, *p* < 0.01), supporting the external evidence of validity of the P Scale among Chinese early adolescents (see Table 6).

**Table 6:** Pearson correlations among P scale, RSES, BMSLSS-general, BMSLSS-global, and loneliness among Chinese early adolescent (sample 2, *N* = 344).

**Constructs**	**P scale**	**RSES**	**Gen-LS**	**Glo-LS**
Positivity (P Scale)	−			
Self-esteem (RSES)	0.60^**^	−		
General Life Satisfaction (BMSLSS-General)	0.44^**^	0.33^**^	−	
Global Life Satisfaction (BMSLSS-Global)	0.42^**^	0.32^**^	0.78^**^	−
Loneliness (ULCA)	−0.60^**^	−0.50^**^	−0.47^**^	−0.42^**^

** *p < 0.01*.

##### Prediction of well-being and depression

Hierarchical multiple regression analyses were applied to estimate the predictive validity of the P scale by assessing the relations between scores on the P Scale at Time 1 and scores on the BASWBSS and DSRSC at Time 2. According to the value of the variance inflation factor (VIF = 1 < 10), none of the variables in the present study suggested issues of multicollinearity, indicating that hierarchical multiple regression analyses were suitable to perform.

First, correlated demographic variables (including gender and age) were entered as the initial block of control variables. Gender was dummy coded as 0 = boys, 1 = girls. Then, scores on the P Scale were entered at the second step of the models. As shown in Table 7, the two overall models accounted for 19.6% and 15.0% of variance respectively. Specifically, scores on the P scale made statistically significant, positive contributions to subjective wellbeing in school (β = 0.43, *p* < 0.001, Δ*R*^2^ = 0.176), and significantly negative contributions to depression at Time 2 (β = −0.34, *p* < 0.001, Δ*R*^2^ = 0.113), revealing evidence of predictive validity for P Scale scores among Chinese early adolescents.

**Table 7:** Hierarchical multiple regression of subjective wellbeing in school and depression at time 2 on the total score of the P scale at time 1 (Sample 3, *N* = 194).

	***B***	***SE***	**β**	***R*^2^**	**Δ*R*^2^**	**ΔF**	***df***
**SUBJECTIVE WELL BEING IN SCHOOL**							
Step 1				0.02	0.02	1.95	(2, 191)
Intercept	12.62	3.44					
Gender^a^	−0.31	0.34	−0.07				
Age	−0.48	0.26	−0.13				
Step 2				0.196	0.176	41.72^***^	(1, 190)
Intercept	2.43	3.50					
Gender^a^	−0.19	0.31	−0.04				
Age	−0.16	0.24	−0.05				
T1 P scale	0.20	0.03	0.43^***^				
**DEPRESSION**							
Step 1				0.037	0.037	3.64^*^	(2, 191)
Intercept	−13.09	9.33					
Gender^a^	1.23	0.91	0.96				
Age	1.74	0.71	0.17^*^				
Step 2				0.150	0.113	25.32^***^	(1, 190)
Intercept	9.23	9.84					
Gender^a^	0.96	0.86	0.08				
Age	1.06	0.69	0.11				
T1 P scale	−0.44	0.09	−0.34^***^				

*P Scale, Positivity scale; T1 P Scale, Total score of the P Scale at Time 1*.

a *0, boys; 1, girls*.

*** *p < 0.001*,

* *p < 0.05*.

#### Measure invariance of the P scale

As in Study 1, three levels of measurement invariance analyses were tested by robust MLR for the assessment of measurement invariance across gender among Chinese early adolescents, and the residuals of items 1 and 4 were correlated in the assessment of measurement invariance as well

Testing the invariance of the P Scale scores across gender, baseline CFA were conducted first for each group (boys and girls) separately. For boys, the model indices were S-B $\chi_{\left(19,\;\text{N}=170\right)}^{2}$ = 35.30, *p* > 0.01; CFI = 0.963; TLI = 0.945; RMSEA = 0.071, 90% CI [0.032, 0.107]; SRMR = 0.043, and for girls, the indices were S-B$\chi_{\left(19,\;\text{N}=174\right)}^{2}$ = 50.05, *p* < 0.01; CFI = 0.945; TLI = 0.919; RMSEA = 0.097, 90% CI [0.065, 0.130]; SRMR = 0.047. In both samples, the one-factor model resulted in an adequate fit. Next, two-group CFAs were conducted to test for comparability of the P Scale scores between boys and girls.

The result showed that both the CFI difference tests and the Satorra-Bentler different test between the configural invariance model and the metric invariance model was ΔCFI = 0.000; ΔS-B$\chi_{\left(7\right)}^{2}$ = 7.04, *p* < 0.01. The indices between metric invariance model and the scalar invariance model was ΔCFI = 0.004; ΔS-B$\chi_{\left(7\right)}^{2}$ = 9.70, *p* < 0.01. The indices suggested that full metric invariance and full scalar invariance were both supported. Using this procedure, a model for scalar invariance across the two gender groups was satisfactory in middle school student. The fit indices for the three invariance models are shown in Table 8.

**Table 8:** Analyses of measurement invariance across gender among early adolescents (Sample 2, *N* = 344).

**Model**	**S-Bχ^2^**	***df***	**CFI**	**TLI**	**RMSEA**	**SMSR**		**ΔS-Bχ^2^(Δ*df*)**	**ΔCFI**
Boys	35.30	19	0.963	0.945	0.071	0.043			
Girls	50.05	19	0.945	0.919	0.097	0.047			
M1:Configural invariance	67.13	38	0.966	0.949	0.067	0.042			
M2:Matric invariance	74.17	45	0.966	0.957	0.061	0.055	M1:M2	7.04(7)	0.000
M3:Scalar invariance	83.87	52	0.962	0.959	0.060	0.059	M2:M3	9.70(7)	−0.004

*ΔS-Bχ^2^, the Satorra-Bentler scaled (mean-adjusted) Chi-square difference between nested models; Δdf, Difference in degrees of freedom between nested models; ΔCFI, Comparative fit index differences between nested models*.

## Disscussion

We conducted two studies to provide empirical evidence of the reliability and validity of the P Scale with Chinese adults and early adolescents. The main findings are summarized below.

First, as expected, both studies supported the hypothesized unidimensional model underlying the P Scale with Chinese individuals. The single-factor solution was obtained using both EFA and CFA methods. In addition, the goodness-of-fit indices for the CFA across both groups were adequate, and the results of the CFA procedures in the two studies both suggested that allowing for a correlation between a pair of error terms (the error of items 1 and 4) would increase model fit. This modification was deemed reasonable given that the content of both items appeared related to a positive view of the future. Furthermore, it is important to note that the error correlations of items 1 and 4 have also been encountered in Italian, American, Spanish, Brazilian, Serbian, Polish and Japanese adults (Caprara et al., 2012b; Heikamp et al., 2014; Borsa et al., 2015), suggesting that the content of the items overlaps to a certain extent across countries (Brown, 2006), and also indicating that the P scale could be simplified by reducing the number of items in future studies.

Second, the reliability of the P scale was supported for Chinese individuals. Across both studies, the Cronbach's alpha coefficients for the eight items exceeded 0.80. Both coefficients exceeded the coefficient (α = 0.75) found with Italian adults (Caprara et al., 2012b), which provided further evidence for the internal consistency reliability of the P scale with Chinese individuals. In addition, the test-retest reliability coefficient showed a strong correlation among Chinese adults, and a moderate correlation among middle school students, providing further support for the notion that POS represents a fundamental disposition, that is, it is a relatively stable characteristic among Chinese adults and middle school students. However, compared with the middle school students, the test-retest reliability coefficient was a little higher for the adults. The reasons for the differences are unclear; however, previous meta-analytic estimates of mean population test-retest correlation coefficients showed that trait consistency increases from childhood to adulthood. Furthermore, the length of the time interval for the retest coefficients displays a negative relation to trait consistency (Roberts and DelVecchio, 2000). Thus, we speculated that this difference might be explained by two similar reasons. First, similar to the aforementioned conclusions of the meta-analysis, the difference in this research might indicate that POS, similar to many personality traits, becomes more stable with increasing maturity. Second, given that the time interval in Study 2 (6 months) was longer than in Study 1 (4 weeks), the lower test-retest coefficient among early adolescents might be influenced by the longer time interval. Both possibilities may operate synergistically to increase the stability of POS reports in older individuals, and future research is needed to explore such possibilities.

Third, we obtained the external evidence of validity of the P scale with Chinese individuals. In both of our studies, we found high positive correlations between P Scale scores and the measures of self-esteem, optimism, and life satisfaction, and moderate negative correlations between P Scale scores and the measures of negative affect and loneliness. The cumulative results also further corroborated the notion that POS can be regarded as a common factor underlying self-esteem, optimism, and life satisfaction, which can be differentiated from other psychological constructs. Furthermore, we provided further evidence that the POS predicts important future outcomes, specifically individuals' reports of subjective well- being and depression for Chinese individuals. Such findings are consistent with the previous finding that individuals with higher P Scale scores were characterized by a pleasant affective state and a low incidence of depressive symptoms (Caprara et al., 2010, 2012b; Alessandri et al., 2012).

Finally, the measurement invariance findings across gender differed for the two groups. For middle school students, the structural, metric and scalar invariance of the P Scale were all generally supported, which indicated that the psychometric properties of the P Scale are quite similar across gender among middle school students. However, for adults, structural invariance, metric invariance and partial scalar invariance were supported, but full scalar invariance was not supported. Specifically, the female sample demonstrated higher values on the intercepts than the male sample on all of the three non-invariant items (2, 3, 5). This difference suggested that response threshold differences for different genders and the effects of systematic responses biases should be considered in the measurement of POS in the Chinese adult population.

To summarize, our research provides some good support for the psychometric properties of the P Scale when used with Chinese adults and early adolescents. This support is important because the P Scale provides a promising, relatively brief measure that can facilitate research in positive psychology and related areas in China as well as other countries. As mentioned in the introduction, research on POS with the P Scale in China remains scarce. Therefore, the applications of the P Scale would make great contribution to the relative area. First, the use of the P Scale could promote the development of numerous studies related to the antecedents and consequences of individual differences in POS across different age levels, such as health, job success, positive interpersonal relationships and other positive outcomes (Caprara et al., 2012a,b). Second, regarded as a basic disposition, POS may play a protective role in human development, buffering against the development of psychological problems as well as promoting optimal levels of functioning in adults and adolescents. All such applications should promote the further development of positive psychology in China. Moreover, these findings obtained within the Chinese context strengthen the argument that POS may be a universal (positive) construct, which had previously been confirmed only in Western countries and one eastern country (e.g., Italy, Spain, United States, Japan).

Nevertheless, there were some important limitations to the present study. First, considering the length of the survey in relation to the anticipated attention span of middle school students, we did not include an optimism measure as in our examination of the external evidence of validity in Study 2. However, the significant correlations between the scores on the P Scale and the RSES and the BMSLSS provide some evidence that the P Scale could effectively measure middle school students' positivity due to the high correlation between optimism and self-esteem, as well as the significant predictive power of optimism in relation to life satisfaction (Chang, 2001; Mäkikangas et al., 2004). Second, given that most of the adults taking part in the Study 1 had earned a university degree and it was somewhat small, the sample may not be representative of the Chinese adult population. Relatedly, our adolescent sample was limited to the middle school student age group. Considering the limitations of both samples, future researchers should make efforts to obtain more representative samples of the full range of adolescents and adults to increase the generalizability of our findings. Furthermore, the Cronbach's alpha of CLOT-R scale (0.63) was relatively low in the current study, which might have unduly reduced the magnitude of the association between the P scale and the CLOT-R. Moreover, studies of measurement invariance across different sub-groups (e.g., age, socioeconomic status) should also be considered to enhance the meaningfulness of the findings. Finally, our study relied exclusively on self-report measures. The use of non-self-report measures (e.g., reports of parents, teachers) to measure POS and related variables in future work should also enhance understanding of the findings.

## Author contributions

All the authors substantially contributed to the conception and the design of the work. LT and DZ participated to the acquisition of data. The first author (LT) prepared the draft and the contributing authors (DZ and EH) reviewed it critically and gave important intellectual content. All the authors worked for the final approval of the version to be published. All the authors are accountable for all the aspects of the work in ensuring that questions related to the accuracy or integrity of any part of the work are appropriately investigated and resolved.

### Conflict of interest statement

The authors declare that the research was conducted in the absence of any commercial or financial relationships that could be construed as a potential conflict of interest.
